# Rapid detection of single nucleotide polymorphisms using the MinION nanopore sequencer: a feasibility study for perioperative precision medicine

**DOI:** 10.1186/s40981-022-00506-7

**Published:** 2022-03-04

**Authors:** Yoshiteru Tabata, Yoshiyuki Matsuo, Yosuke Fujii, Atsufumi Ohta, Kiichi Hirota

**Affiliations:** 1 Department of Human Stress Response Science, Institute of Biomedical Science, Kansai Medical University, Hirakata, Osaka Japan; 2 Department of Anesthesia, Otsu City Hospital, Otsu, Shiga Japan

**Keywords:** Genotyping, Nanopore sequencing, Perioperative management, Precision medicine, Single nucleotide polymorphisms

## Abstract

**Introduction:**

Precision medicine is a phrase used to describe personalized medical care tailored to specific patients based on their clinical presentation and genetic makeup. However, despite the fact that several single nucleotide polymorphisms (SNPs) have been reported to be associated with increased susceptibility to particular anesthetic agents and the occurrence of perioperative complications, genomic profiling and thus precision medicine has not been widely applied in perioperative management.

**Methods:**

We validated six SNP loci known to affect perioperative outcomes in Japanese patients using genomic DNA from saliva specimens and nanopore sequencing of each SNP loci to facilitate allele frequency calculations and then compared the nanopore results to those produced using the conventional dideoxy sequencing method.

**Results:**

Nanopore sequencing reads clustered into the expected genotypes in both homozygous and heterozygous cases. In addition, the nanopore sequencing results were consistent with those obtained using conventional dideoxy sequencing and the workflow provided reliable allele frequency estimation, with a total analysis time of less than 4 h.

**Conclusion:**

Thus, our results suggest that nanopore sequencing is a promising and versatile tool for SNP genotyping, allowing for rapid and feasible risk prediction of perioperative outcomes.

**Supplementary Information:**

The online version contains supplementary material available at 10.1186/s40981-022-00506-7.

## Introduction

The most critical role of any anesthesiologist is to ensure the safety of their patient during the perioperative period and provide the skills and expertise to ensure a smooth surgical experience. A preoperative medical assessment is used to ensure the safety and comfort of patients undergoing surgery. However, even with preoperative testing, it is difficult to predict individual susceptibility to particular anesthetic drugs and the risk of potential postoperative complications such as postoperative nausea and vomiting [1, 2].

Precision medicine is an emerging approach designed to facilitate improved prevention and treatment of human diseases [3]. This approach uses personalized medical care tailored to a specific patient based on their clinical presentation and genetic background to improve clinical outcomes.

Previous studies have shown that there are a range of specific genetic variations associated with changes in susceptibility to specific anesthetic agents and perioperative outcomes [4]. Single nucleotide variations (SNV) are characterized by an alteration at a single position within a DNA sequence and when this SNV is present in at least 1% of the population, it is referred to as a single nucleotide polymorphism (SNP). Along with recent advances in DNA sequencing technology, the development of an integrated SNP dataset would be beneficial for the prediction of potential perioperative risk and the management of anesthetic complications.

Given this, we designed this study to evaluate a streamlined workflow for SNV/SNP genotyping using nanopore sequencing technology, with a view to enabling genetic-based perioperative risk evaluations in preoperative patients. The MinION nanopore sequencer is a portable DNA/RNA sequencing platform that provides on-site genetic analysis with rapid and affordable deployment [5, 6]. We developed a simple bioinformatics pipeline to reliably detect several SNPs of interest within the nanopore sequencing data. We selected six SNP loci affecting perioperative outcomes for validation, and the utility of the workflow was evaluated using SNP allele frequency estimations and their comparison with more conventional sequencing technologies.

## Methods

### Samples and DNA extraction

This study was approved by the institutional review board at Kansai Medical University Hospital (No. 2020285, February 19, 2021) and saliva samples were collected from five willing participants. Approximately 1 mL of saliva was mixed with 4 mL of phosphate-buffered saline (PBS, Nacalai Tesque, Kyoto, Japan) and centrifuged at 1800×*g* for 5 min before being resuspended in 0.3 mL of PBS and subjected to DNA extraction using the Maxwell RSC Blood DNA Kit (AS1400, Promega, Madison, WI, USA) on the Maxwell RSC automated nucleic acid purification platform (AS4500, Promega). Briefly, the sample (0.3 mL) was mixed with lysis buffer (0.3 mL) and proteinase K solution (30 μL), incubated at 56 °C for 20 min, and transferred to a Maxwell RSC Cartridge for magnetic bead-based DNA extraction. DNA was eluted in 50 μL elution buffer and quantified using the QuantiFluor ONE dsDNA System (E4871, Promega).

### DNA amplification

A detailed protocol of the 2-step polymerase chain reaction (PCR) for nanopore amplicon sequencing is available at protocols.io (10.17504/protocols.io.bwr5pd86). Briefly, six SNP loci were used for amplicon sequencing (Table 1) and the locus-specific sequences of the PCR primers are listed in Table 2.

**Table 1:** List of SNPs evaluated in this study

SNP	Gene	Full gene name	Clinical significance	Ref.
rs1045642	*ABCB1*	ATP binding cassette subfamily B member 1	A>G: increased susceptibility to PONV A>G: increased opioid dose requirement	[7, 8]
rs1799971	*OPRM1*	Opioid receptor mu 1	A>G: increased opioid dose requirement	[9–11]
rs2165870	*CHRM3*	Cholinergic receptor muscarinic 3	A>G: decreased susceptibility to PONV	[12, 13]
rs4369876	*SCN9A*	Sodium voltage-gated channel alpha subunit 9	C>A: decreased pain sensitivity	[14]
rs33985936	*SCN11A*	Sodium voltage-gated channel alpha subunit 11	C>T: increased pain sensitivity	[15]
rs140124801	*KCNG4*	Potassium voltage-gated channel modifier subfamily G member 4	C>T: decreased pain sensitivity	[16]

*PONV* postoperative nausea and vomiting

**Table 2:** Primer and probe sequences used in the nanopore-mediated genotyping of the six target SNPs

SNP	Locus-specific primer sequence (5′ to 3′) F, forward; R, reverse		PCR product (bp)	Probe sequence
rs1045642	F: R:	ACTAACCCAAACAGGAAGTGTGG GTGTGCTGGTCCTGAAGTTGA	413	CCTCACNATCTCT
rs1799971	F: R:	AAGGTGGGAGGGGGCTATAC ACTTCTCTGCTCCTGAAATTTTGAA	738	TAGATGGCNACCT
rs2165870	F: R:	AGCTAATGCAGCTACTAGTTAA TGCTATACATCACATCCTCAAGT	927	AGCCTGNTATACT
rs4369876	F: R:	TTTGTCCACGCTGCTTCCAAAAC TGCTGGTTTGTATTGTGGCCT	438	TTTCANATAATTT
rs33985936	F: R:	TGGGTATCAAAGGGCAGCCA AGCACTGGATCGATTCCGCC	466	CATGCCTGANGCC
rs140124801	F: R:	CACCAGGTGGTCTATGCGGG TACGTGGCCGAGAAGGAGTC	481	CGTAGCCCANCGT

The locus-specific inner primer pairs used in the first PCR contained the following 5′ tail sequences: forward, TTTCTGTTGGTGCTGATATTGC + locus-specific sequence (F); reverse, ACTTGCCTGTCGCTCTATCTTC + locus-specific sequence (R). The base corresponding to the SNP site in each probe is represented by an “N” in each probe sequence

A total of 20 ng of saliva DNA was used as a template to amplify the target genomic region and the locus-specific inner primers used in the first PCR each included the following 5′ tail sequences: forward 5′-TTTCTGTTGGTGCTGATATTGC - locus-specific sequence-3′; reverse 5′-ACTTGCCTGTCGCTCTATCTTC - locus-specific sequence-3′. PCR amplification was performed using Platinum II Hot-Start PCR Master Mix (14000012, Thermo Fisher Scientific, Waltham, MA, USA) with 0.2 μM of each inner primer in a total volume of 25 μL. Amplification conditions were as follows: initial denaturation at 94 °C for 2 min, 35 cycles of 94 °C for 15 s, 60 °C for 15 s, 68 °C for 30 s, followed by a final extension at 68 °C for 1 min. The resultant amplicons (1 μL) were subjected to a second PCR to introduce the indices (barcodes) and the 5′ tags required for adapter attachment. These reaction mixtures (25 μL) contained KAPA2G Robust HotStart ReadyMix (KK5701, KAPA Biosystems, Wilmington, MA, USA) and the barcoded outer primers (0.5 μL) supplied in the PCR Barcoding Kit (SQK-PBK004, Oxford Nanopore Technologies, Oxford, UK). Cycling conditions were as follows: initial denaturation at 95 °C for 3 min; 15 cycles of 95 °C for 15 s, 62 °C for 15 s, 72 °C for 30 s, followed by a final extension at 72 °C for 1 min. Amplified DNA was then purified using AMPure XP (A63880, Beckman Coulter, Brea, CA, USA) and quantified using a QuantiFluor ONE dsDNA System.

### Nanopore sequencing

The purified barcoded amplicons were then pooled, and 100 fmol was applied as template in the library preparation which was completed using the PCR Barcoding Kit. These libraries were loaded onto the R9.4.1 flow cell (FLO-MIN106, Oxford Nanopore Technologies) and sequenced on the MinION Mk1C with MinKNOW software version 21.05.21 (Oxford Nanopore Technologies). Base-calling was performed in real time via Guppy version 5.0.13 (Oxford Nanopore Technologies) using the following settings: fast basecalling model, trim_barcodes=on, require_barcodes_both_ends=off, detect_mid_strand_barcodes=on, min_score=60. Called reads (FASTQ format) were filtered to generate pass reads, with a minimum Phred quality score of 8.

### DNA sequencing using the dideoxy method

The first round PCR products described above were purified using an AMPure XP and sequenced on both strands using the following primers: forward TTTCTGTTGGTGCTGATATTGC and reverse ACTTGCCTGTCGCTCTATCTTC, which correspond with the 5′ tails described above. Sequencing was performed on a 3130xl Genetic Analyzer using a BigDye Terminator v3.1 Cycle Sequencing kit (Thermo Fisher Scientific).

### Bioinformatic analysis

The average Phred quality scores of the nanopore sequencing reads were analyzed using NanoPlot ver. 1.27.0 and the allele frequencies for each of the target SNP sites were determined using the following bioinformatics pipeline in SeqKit version 0.13.2. Step 1: Reads with a perfect match to the probe sequence specific to each SNP site (Table 2) were extracted and saved into individual files. Where this command identifies the probe sequence (SNP site is represented by “N”) in both the forward and reverse strands of the amplicon. Step 2: random sampling of one thousand reads per target. Step 3: Reads were sorted by SNP genotype, and allele frequencies were calculated based on the read count. The exact commands used to generate this data are provided in Additional file 1.

## Results

Variant data were obtained from the Integrated Genome Variation Database, TogoVar [17] (Additional file 2: Table S1) and then used to identify six SNP loci associated with specific perioperative outcomes that could be used to evaluate the feasibility of nanopore sequencing in the real-time analysis of genetic predisposition to adverse perioperative outcomes (Table 1). Genomic DNA was extracted from the saliva of five individuals and then subjected to PCR amplification of the target loci using specific primers (Table 2), before sequencing on the MinION platform. These amplicons were also sequenced using the dideoxy method to verify the genotypes (Additional file 3, Figs. S1–S6).

**Fig. 1:**
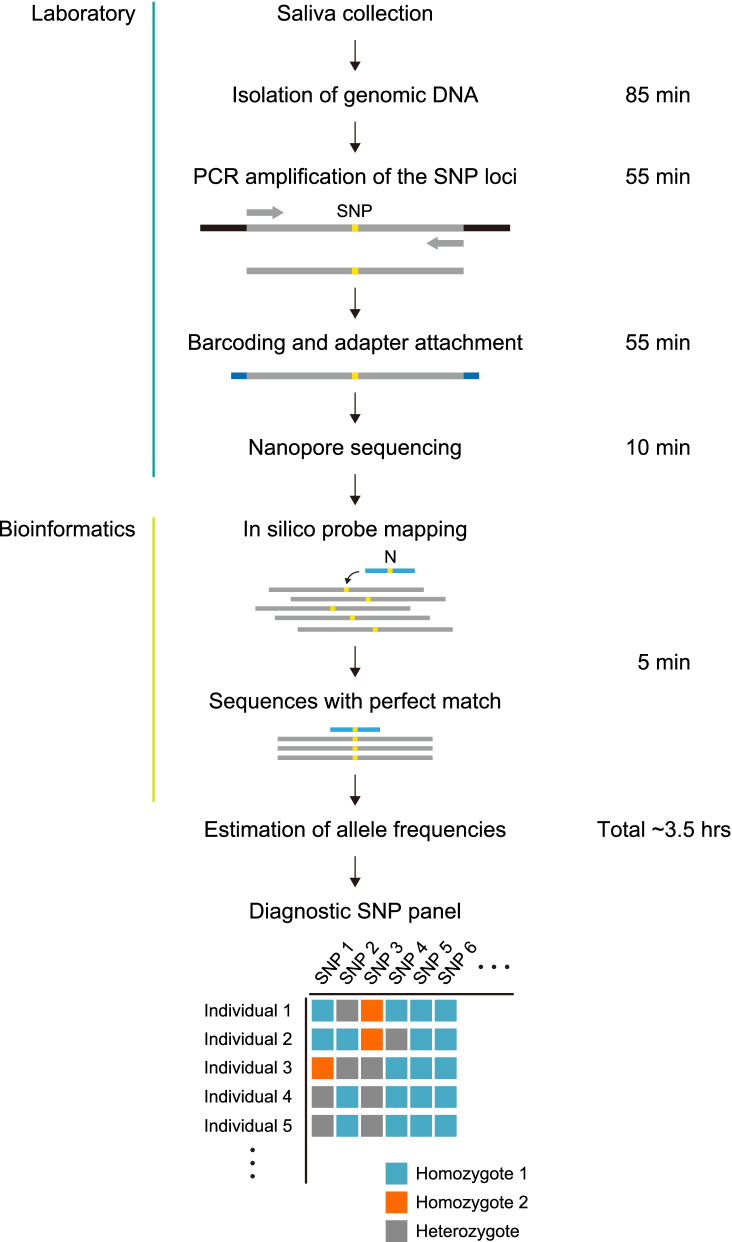
Schematic describing the laboratory and bioinformatics pipelines used to complete targeted SNP genotyping using the nanopore sequencing technology

Nanopore sequencing was performed on up to 12 barcoded samples at a time, and 10-min of MinION sequencing yielded an average of over 30,000 pass reads making this sequencing time sufficient for SNP genotyping using our bioinformatic pipeline. Nanopore sequencing reads with a perfect match to the probe sequence (Table 2) were sorted, and 1000 reads per sample were collected and then used to determine the allele frequencies of each SNP site (Additional file 4: Table S2). The estimation of the allele frequencies for the target SNP loci was completed in approximately 3.5 h (Fig. 1).

**Fig. 2:**
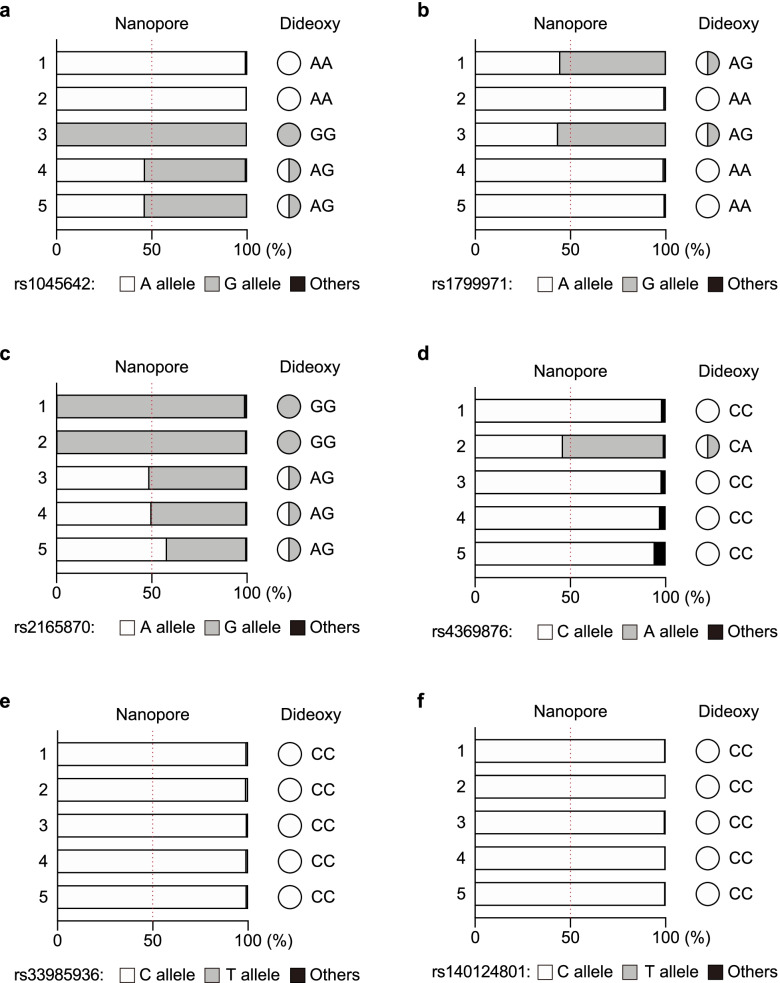
Genotyping of SNPs associated with perioperative outcomes and anesthetic complications. Six SNP loci were genotyped from five individuals: **a** rs1045642, **b** rs1799971, **c** rs2165870, **d** rs4369876, **e** rs33985936, **f** rs140124801. The graphs show the allele frequencies determined by nanopore amplicon sequencing. These amplicons were then sequenced using traditional dideoxy sequencing to confirm their genotypes

Allele frequencies as determined by nanopore sequencing fluctuated around the theoretical value of 50% for heterozygote variants. While in the homozygote samples, the reads were binned into only one of the given variants, with allele frequencies of approximately 100% (Fig. 2). A maximum deviation of 8.4% from the expected value was observed (Fig. 2c and Additional file 5, Table S5) and only a small fraction of false-positive variants were detected (< 6%; Fig. 2c and Additional file 5: Tables S3–S8). Given that the mean Phred quality score of the nanopore reads was 12.2 (~ 94% accuracy, Additional file 4: Table S2), we suggest that such spurious sequence variation is likely the result of minor sequencing errors. Taken together, our results show that the nanopore sequencing reads are reasonably well clustered within the expected allele frequencies, demonstrating the reliability of this approach for SNP genotyping.

## Discussion

Recent technological advances in genomic research, including next-generation sequencing (NGS), have enabled rapid and precise analysis of large amounts of genomic data [18]. This has meant that genomic profiling is increasingly being used to identify subpopulations with different susceptibilities to particular drugs and medical treatments, facilitating better, more personalized treatment [19]. These integrated genomic datasets demonstrate significant potential for developing targeted medical strategies based on individual variability in specific patient characteristics. Here, we developed a novel nanopore-based sequencing strategy for rapid SNP genotyping and evaluated its feasibility for genomic profiling in the context of perioperative care, in an attempt to integrate precision medicine into this unique medical discipline.

Recent reports have described several genetic variations that may affect a patient’s susceptibility to particular anesthetic agents and the occurrence of perioperative complications [4]. These include SNP rs1799971 located in the gene encoding opioid receptor mu 1 (*OPRM1*), which has been extensively studied as an example of a genetic variant affecting perioperative outcomes. The μ-opioid receptor is the primary site of action for most perioperative opioids, including morphine and fentanyl [20]. This SNP is located in the protein-coding region, and the 118A>G substitution causes an amino acid change (p.Asn40Asp), potentially affecting receptor expression and the downstream signaling pathway [21, 22]. It has also been suggested that patients with AG and GG genotypes require increased opioid doses for pain management [9–11]; however, the mechanisms underlying these potential associations remain unclear. In addition, while the overall frequency of the G allele for rs1799971 has been reported to be 12.2%, there are considerable differences between ethnicities, with Asian populations often demonstrating an increased alternate G allele frequency (approximately 40%; TogoVarID: tgv27548008, Additional file 2: Table S1). This was supported by the fact that the G allele frequency for rs1799971 in Japanese samples ranges from 42 to 44.7% (Additional file 2: Table S1). Here, we detected the G allele in two of the five Japanese study participants, which was consistent with previous observations. SNPs rs1045642 [7, 8] and rs2165870 [12, 13] also experience a moderate-to-high alternate allele frequency in the Japanese population, with frequencies of approximately 60% and 70%, respectively. This was consistent with our nanopore-based SNP genotyping which identified an alternate G allele for rs1045642 in three of the five Japanese participants and the rs2165870 alternate G allele in all five samples. In contrast to these findings, public datasets suggest that the alternate allele frequencies for rs4369876 [14], rs33985936 [15], and rs140124801 [16] are quite low. This was also the case in our study where all but one individual, with the alternate A allele for rs4369876, presented as homozygous for the major reference allele at all three SNP loci. Thus, taken together, these results suggest that nanopore-mediated SNP genotyping accurately reflects the common allele frequencies associated with these SNPs in the Japanese population, even in this very limited sample.

Conventional NGS generates vast quantities of highly accurate sequencing data on a large scale [18, 23]. However, the cost-effectiveness of these platforms are dependent on the acquisition and evaluation of large numbers of samples to be analyzed in one batch. Nanopore sequencing enables the real-time processing of a small number of samples on a case-by-case basis, making this analysis more suitable for genetic testing in personalized clinical care as opposed to large-scale clinical studies [24, 25]. Given these features, we designed this study to establish and evaluate a workflow for rapid SNP genotyping on a per patient basis, and although this study focused on SNPs linked to perioperative outcomes, nanopore amplicon sequencing, and a similar bioinformatics pipeline could easily be applied in the detection of a wide variety of genetic variations.

Despite the obvious advantages presented by this approach, our study did suffer from several limitations. First, the reliability of this method was only evaluated for a very small number of known SNPs in a very small population. Second, this genotyping workflow limits the identification of novel or unknown SNPs. Third, the clinical relevance of each SNP has not yet been thoroughly validated. Accumulating evidence has shown the potential of several SNPs to act as genetic markers for risk prediction around perioperative outcomes. However, the utility of SNP genotyping data in anesthetic management remains unclear. Further studies are required to elucidate the underlying mechanisms linking these genetic variations to the clinical phenotype and to establish a basis for the practice of precision medicine in perioperative care.

## Conclusion

Nanopore sequencing reads were reasonably well clustered within their expected genotypes, and this simplified workflow provided reliable allele frequency estimation with a total analysis time of less than 4 h. This suggests that nanopore sequencing may be a promising and versatile tool for SNP genotyping, allowing for rapid and reliable perioperative risk prediction in clinical settings.

## Supplementary Information

**Additional file 1:.** Bioinformatics analysis.

**Additional file 2: Table S1.** Allele frequency data for selected SNPs associated with perioperative outcomes.

**Additional file 3: Figures S1-S6.** SNP genotyping using dideoxy sequencing.

**Additional file 4: Table S2.** Statistics of the nanopore sequencing data.

**Additional file 5: Tables S3-S8.** Allele frequencies for each SNP as determined by nanopore sequencing.

## Data Availability

The data used in this study is available from the corresponding author upon reasonable request.

## Abbreviations

NGS: Next-generation sequencing
PCR: Polymerase chain reaction
SNP: Single nucleotide polymorphism
SNV: Single nucleotide variation

## References

[1] Macario A, Weinger M, Carney S, Kim A (1999). Which clinical anesthesia outcomes are important to avoid? The perspective of patients. Anesth Analg.

[2] Watcha MF, White PF (1992). Postoperative nausea and vomiting. Its etiology, treatment, and prevention. Anesthesiology..

[3] Jameson JL, Longo DL (2015). Precision medicine--personalized, problematic, and promising. N Engl J Med.

[4] Kumar S, Kundra P, Ramsamy K, Surendiran A (2019). Pharmacogenetics of opioids: a narrative review. Anaesthesia..

[5] Kono N, Arakawa K (2019). Nanopore sequencing: Review of potential applications in functional genomics. Dev Growth Differ.

[6] Kai S, Matsuo Y, Nakagawa S, Kryukov K, Matsukawa S, Tanaka H (2019). Rapid bacterial identification by direct PCR amplification of 16S rRNA genes using the MinION nanopore sequencer. FEBS Open Bio.

[7] Choi EM, Lee MG, Lee SH, Choi KW, Choi SH (2010). Association of ABCB1 polymorphisms with the efficacy of ondansetron for postoperative nausea and vomiting. Anaesthesia..

[8] Lotsch J, von Hentig N, Freynhagen R, Griessinger N, Zimmermann M, Doehring A (2009). Cross-sectional analysis of the influence of currently known pharmacogenetic modulators on opioid therapy in outpatient pain centers. Pharmacogenet Genomics.

[9] Chou WY, Yang LC, Lu HF, Ko JY, Wang CH, Lin SH (2006). Association of mu-opioid receptor gene polymorphism (A118G) with variations in morphine consumption for analgesia after total knee arthroplasty. Acta Anaesthesiol Scand.

[10] Chou WY, Wang CH, Liu PH, Liu CC, Tseng CC, Jawan B (2006). Human opioid receptor A118G polymorphism affects intravenous patient-controlled analgesia morphine consumption after total abdominal hysterectomy. Anesthesiology..

[11] Reyes-Gibby CC, Shete S, Rakvag T, Bhat SV, Skorpen F, Bruera E (2007). Exploring joint effects of genes and the clinical efficacy of morphine for cancer pain: OPRM1 and COMT gene. Pain..

[12] Klenke S, de Vries GJ, Schiefer L, Seyffert N, Bachmann HS, Peters J (2018). CHRM3 rs2165870 polymorphism is independently associated with postoperative nausea and vomiting, but combined prophylaxis is effective. Br J Anaesth.

[13] Janicki PK, Vealey R, Liu J, Escajeda J, Postula M, Welker K (2011). Genome-wide Association study using pooled DNA to identify candidate markers mediating susceptibility to postoperative nausea and vomiting. Anesthesiology..

[14] Duan G, Xiang G, Zhang X, Yuan R, Zhan H, Qi D (2013). A single nucleotide polymorphism in SCN9A may decrease postoperative pain sensitivity in the general population. Anesthesiology..

[15] Sun J, Duan G, Li N, Guo S, Zhang Y, Ying Y (2017). SCN11A variants may influence postoperative pain sensitivity after gynecological surgery in Chinese Han female patients. Medicine (Baltimore).

[16] Lee MC, Nahorski MS, Hockley JRF, Lu VB, Ison G, Pattison LA (2020). Human labor pain is influenced by the voltage-gated potassium channel KV6.4 Subunit. Cell Rep.

[17] TogoVar [Internet]. Tokyo: Japan Science and Technology Agency (Japan), National Bioscience Database Center; 2018 Available from: https://togovar.biosciencedbc.jp. Accessed 18 Dec 2021.

[18] Goodwin S, McPherson JD, McCombie WR (2016). Coming of age: ten years of next-generation sequencing technologies. Nat Rev Genet.

[19] Frampton GM, Fichtenholtz A, Otto GA, Wang K, Downing SR, He J (2013). Development and validation of a clinical cancer genomic profiling test based on massively parallel DNA sequencing. Nat Biotechnol.

[20] Mestek A, Hurley JH, Bye LS, Campbell AD, Chen Y, Tian M (1995). The human mu opioid receptor: modulation of functional desensitization by calcium/calmodulin-dependent protein kinase and protein kinase C. J Neurosci.

[21] Bond C, LaForge KS, Tian M, Melia D, Zhang S, Borg L (1998). Single-nucleotide polymorphism in the human mu opioid receptor gene alters beta-endorphin binding and activity: possible implications for opiate addiction. Proc Natl Acad Sci U S A.

[22] Zhang Y, Wang D, Johnson AD, Papp AC, Sadee W (2005). Allelic expression imbalance of human mu opioid receptor (OPRM1) caused by variant A118G. J Biol Chem.

[23] Nielsen R, Paul JS, Albrechtsen A, Song YS (2011). Genotype and SNP calling from next-generation sequencing data. Nat Rev Genet.

[24] Tanaka H, Matsuo Y, Nakagawa S, Nishi K, Okamoto A, Kai S (2019). Real-time diagnostic analysis of MinION-based metagenomic sequencing in clinical microbiology evaluation: a case report. JA Clin Rep.

[25] Matsuo Y, Komiya S, Yasumizu Y, Yasuoka Y, Mizushima K, Takagi T (2021). Full-length 16S rRNA gene amplicon analysis of human gut microbiota using MinION nanopore sequencing confers species-level resolution. BMC Microbiol.

