# Supplementary material for: Rapid detection of single nucleotide polymorphisms using the MinION nanopore sequencer: a feasibility study for perioperative precision medicine
Source: JA Clin Rep. 2022 Mar 4;8:17. doi: 10.1186/s40981-022-00506-7 (PMC8897523; doi:10.1186/s40981-022-00506-7)
Supplement: Supplementary file 1 — Additional file 1:. Bioinformatics analysis. [file 40981_2022_506_MOESM1_ESM.pdf]

Bioinformatics analysis

The exact commands used to generate data

Step 1 Extraction of reads with a perfect match to the probe sequence

- Input: seq.fastq (pass reads)
- Output: seq_1.fastq

```
seqkit grep -s -d -p (probe sequence) seq.fastq > seq_1.fastq
```

Step 2 Random sampling (1000 reads)

- Input: seq_1.fastq
- Output: seq_2.fastq

```
seqkit sample -n 1000 -2 seq_1.fastq > seq_2.fastq
```

Step 3 Estimation of allele frequencies

- Input: seq_2.fastq
- Output: read counts

```
seqkit grep -s -d -p (allele sequence) seq_2.fastq | seqkit seq -n | wc -l
```

Sequence information

rs1045642

probe sequence: CCTCACNATCTCT
reference allele: CCTCACAATCTCT
alternate allele: CCTCACGATCTCT
others: CCTCACYATCTCT

rs1799971

probe sequence: TAGATGGCNACCT
reference allele: TAGATGGCAACCT
alternate allele: TAGATGGCGACCT
others: TAGATGGCYACCT

rs2165870

probe sequence: AGCCTGNTATACT
reference allele: AGCCTGATATACT
alternate allele: AGCCTGGTATACT
others: AGCCTGYTATACT

rs4369876

probe sequence: TTTCANATAATTT
reference allele: TTTCACATAATTT
alternate allele: TTTCAAATAATTT
others: TTTCAKATAATTT

rs33985936

probe sequence: CATGCCTGANGCC
reference allele: CATGCCTGACGCC
alternate allele: CATGCCTGATGCC
others: CATGCCTGARGCC

rs140124801

probe sequence: CGTAGCCCANCGT
reference allele: CGTAGCCCACCGT
alternate allele: CGTAGCCCATCGT
others: CGTAGCCCARCGT

Example

For genotyping of SNP rs1045642:

Step 1 Extraction of reads with a perfect match to the probe sequence

```
seqkit grep -s -d -p CCTCACNATCTCT seq.fastq > seq_1.fastq
```

Step 2 Random sampling (1000 reads)

```
seqkit sample -n 1000 -2 seq_1.fastq > seq_2.fastq
```

Step 3 Estimation of allele frequencies

Reference allele (A):

```
seqkit grep -s -d -p CCTCACAATCTCT seq_2.fastq | seqkit seq -n | wc -l
```

Alternate allele (G):

```
seqkit grep -s -d -p CCTCACGATCTCT seq_2.fastq | seqkit seq -n | wc -l
```

Others (C/T):

```
seqkit grep -s -d -p CCTCACYATCTCT seq_2.fastq | seqkit seq -n | wc -l
```
