# Supplementary material for: Rapid detection of single nucleotide polymorphisms using the MinION nanopore sequencer: a feasibility study for perioperative precision medicine
Source: JA Clin Rep. 2022 Mar 4;8:17. doi: 10.1186/s40981-022-00506-7 (PMC8897523; doi:10.1186/s40981-022-00506-7)
Supplement: Supplementary file 2 — Additional file 2: Table S1. Allele frequency data for selected SNPs associated with perioperative outcomes. [file 40981_2022_506_MOESM2_ESM.pdf]

Table S1 Allele frequency data for selected SNPs associated with perioperative outcomes

SNP	Variant	Alternate allele frequency (%)					
	Ref>Alt	Japanese genetic variation database				gnomAD Genomes	
		GEM-J	JGA-SNP	ToMMo	HGVD	East Asian	Total
rs1045642	A>G	58.3	57.7	58.3	59.9	63.1	56.0
rs1799971	A>G	44.3	44.7	44.7	42.0	33.7	12.2
rs2165870	A>G	74.1	NA	73.7	NA	84.6	75.6
rs4369876	C>A	4.5	4.4	4.4	5.3	5.7	1.0
rs33985936	C>T	8.5	8.4	8.2	9.3	12.5	20.6
rs140124801	C>T	NA	NA	NA	NA	0	0.5

Ref: reference allele, Alt: alternate allele, NA: not available. Data are from the integrated genome variation database TogoVar[1]. Allele frequency data of Japanese population from the public databases (GEM-J[2], JGA-SNP[3], ToMMo[4], and HGVD[5]) are shown. The data for east Asian and total populations (African-American/African, Ashkenazi Jewish, East Asian, Finnish, Latino, Non-Finnish European, and Other) are obtained from gnomAD[6].

1. TogoVar: Tokyo: Japan Science and Technology Agency (Japan), National Bioscience Database Center. TogoVar ID tgv30006530 (rs1045642), tgv27548008 (rs1799971), tgv5354698 (rs2165870), tgv9362554 (rs4369876), tgv12122442 (rs33985936), tgv56421148 (rs140124801); [2021/12/18]. Available from: https://togovar.biosciencedbc.jp
2. GEM-J: "GEM Japan Whole Genome Aggregation (GEM-J WGA) Panel". Japan: GEnome Medical alliance Japan Project (GEM-J). Available from: https://togovar.biosciencedbc.jp/doc/datasets/gem_j_wga
3. JGA-SNP: Variant set aggregated from SNP-chip data in NBDC Human Database/JGA. Tokyo: Japan Science and Technology Agency (Japan), National Bioscience Database Center. JGA-SNP dataset; [2021/12/18]. Available from: https://togovar.biosciencedbc.jp/doc/datasets/jga_ngs
4. ToMMo: Tohoku Medical Megabank Organization 8.3K JPN Allele Frequency Panel.
Tadaka et al., "jMorp updates in 2020: large enhancement of multi-omics data resources on the general Japanese population", Nucleic Acids Research. 2021 Jan 8;49(D1):D536-D544. https://doi.org/10.1093/nar/gkaa1034
5. HGVD: The Human Genetic Variation Database.
K. Higasa et al. "Human genetic variation database, a reference database of genetic variations in the Japanese population" J Hum Genet 61:547-553, 2016. https://doi.org/10.1038/jhg.2016.12
6. gnomAD: The Genome Aggregation Database. Karczewski, K.J., Francioli, L.C., Tiao, G. et al. The mutational constraint spectrum quantified from variation in 141,456 humans. Nature 581, 434–443 (2020). https://doi.org/10.1038/s41586-020-2308-7
