# Supplementary material for: Rapid detection of single nucleotide polymorphisms using the MinION nanopore sequencer: a feasibility study for perioperative precision medicine
Source: JA Clin Rep. 2022 Mar 4;8:17. doi: 10.1186/s40981-022-00506-7 (PMC8897523; doi:10.1186/s40981-022-00506-7)
Supplement: Supplementary file 3 — Additional file 3: Figures S1-S6. SNP genotyping using dideoxy sequencing. [file 40981_2022_506_MOESM3_ESM.pdf]

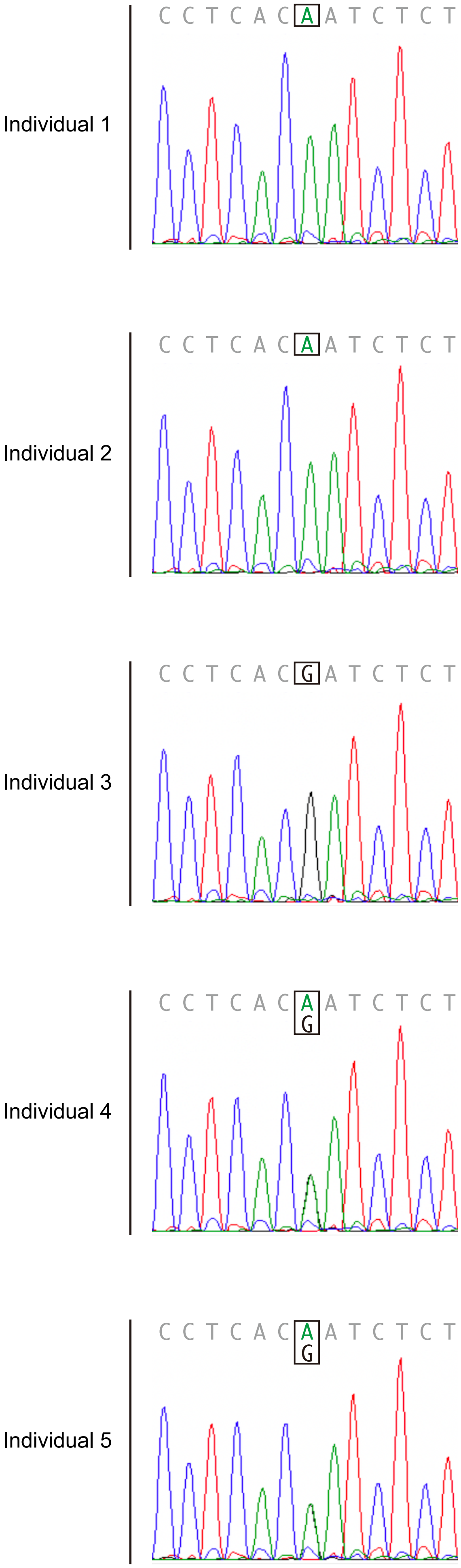

Fig. S1 Genotyping of SNP rs1045642 by the dideoxy sequencing. Representative electropherograms of five individuals are shown: 1, A/A homozygote; 2, A/A homozygote; 3, G/G homozygote; 4, A/G heterozygote; 5, A/G heterozygote. The bases corresponding to the SNP site are boxed.

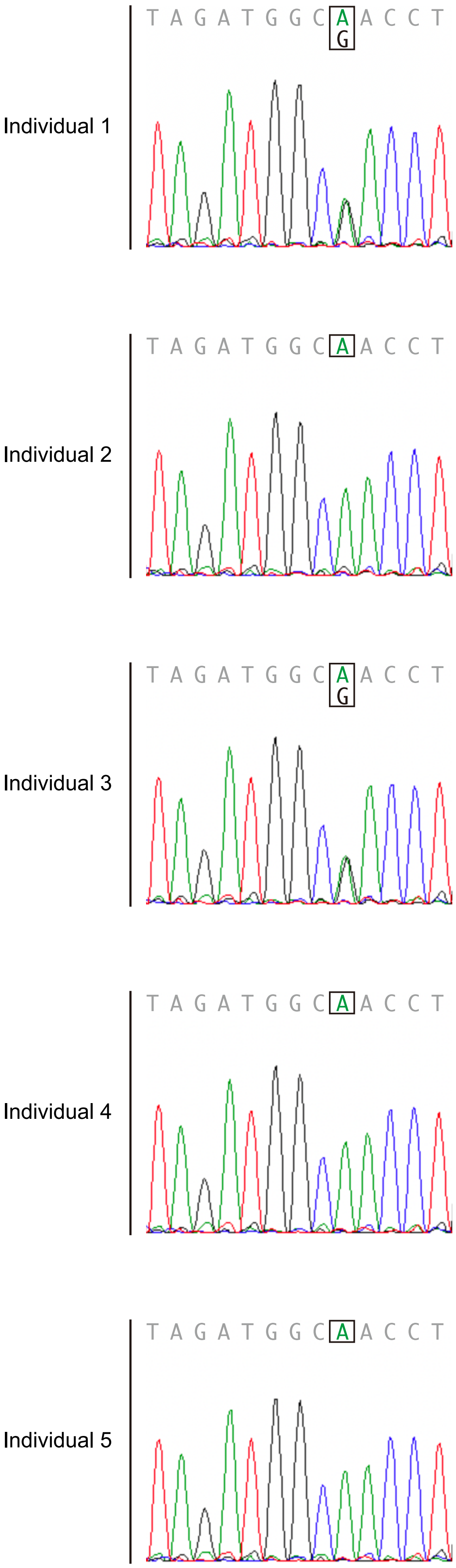

Fig. S2 Genotyping of SNP rs1799971 by the dideoxy sequencing. Representative electropherograms of five individuls are shown: 1, A/G heterozygote; 2, A/A homozygote; 3, A/G heterozygote; 4, A/A homozygote; 5, AA homozygote. The bases corresponding to the SNP site are boxed.

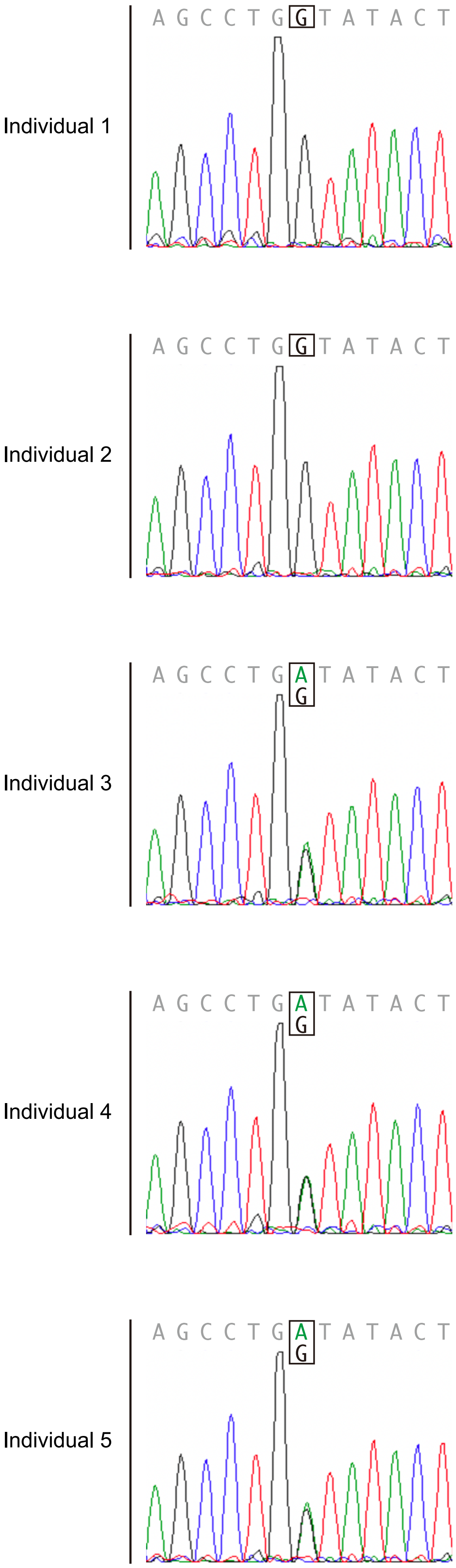

Fig. S3 Genotyping of SNP rs2165870 by the dideoxy sequencing. Representative electropherograms of five individuals are shown: 1, G/G homozygote; 2, G/G homozygote; 3, A/G heterozygote; 4, A/G heterozygote; 5, A/G heterozygote. The bases corresponding to the SNP site are boxed.

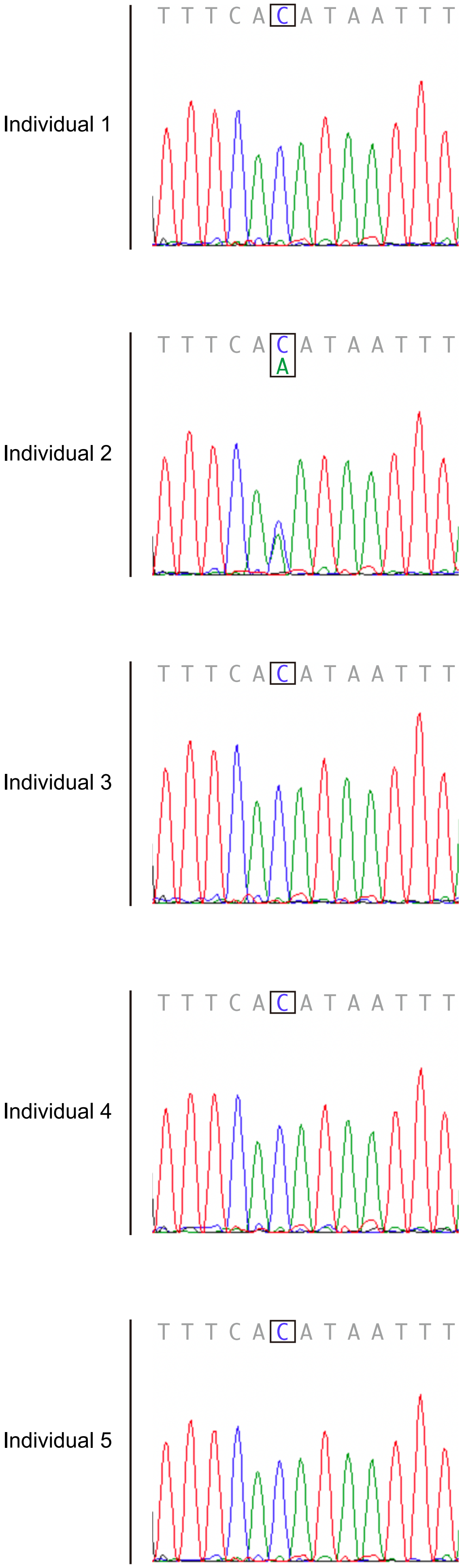

Fig. S4 Genotyping of SNP rs4369876 by the dideoxy sequencing. Representative electropherograms of five individuals are shown: 1, C/C homozygote; 2, C/A heterozygote; 3, C/C homozygote; 4, C/C homozygote; 5, C/C homozygote. The bases corresponding to the SNP site are boxed.

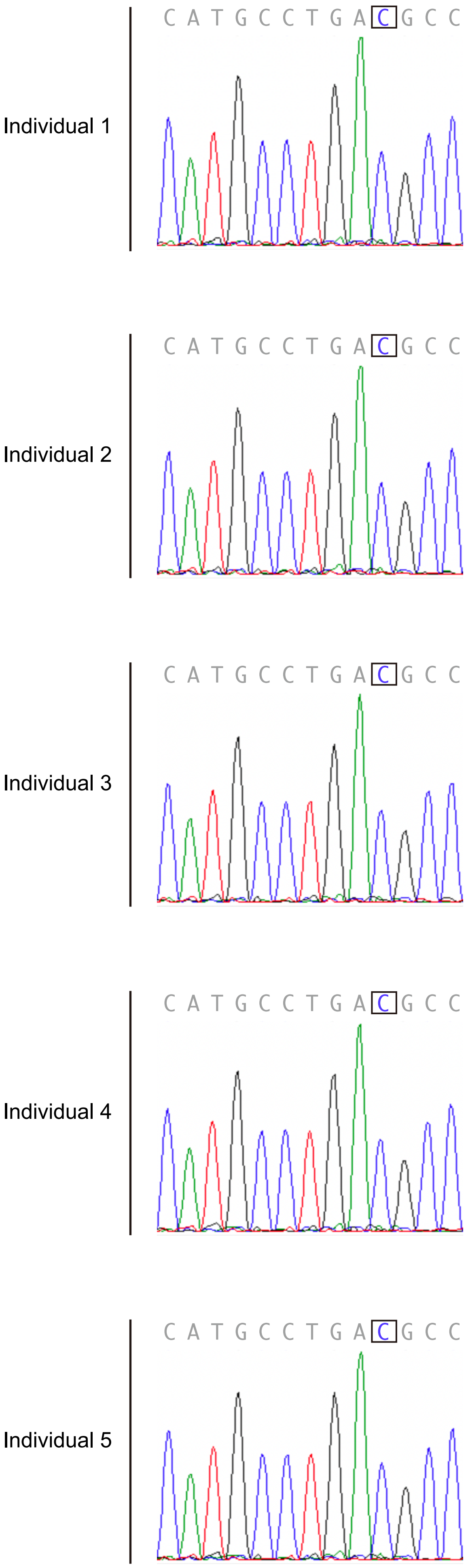

Fig. S5 Genotyping of SNP rs33985936 by the dideoxy sequencing. Representative electropherograms of five individuals are shown: 1, C/C homozygote; 2, C/C homozygote; 3, C/C homozygote; 4, C/C homozygote; 5, C/C homozygote. The bases corresponding to the SNP site are boxed.

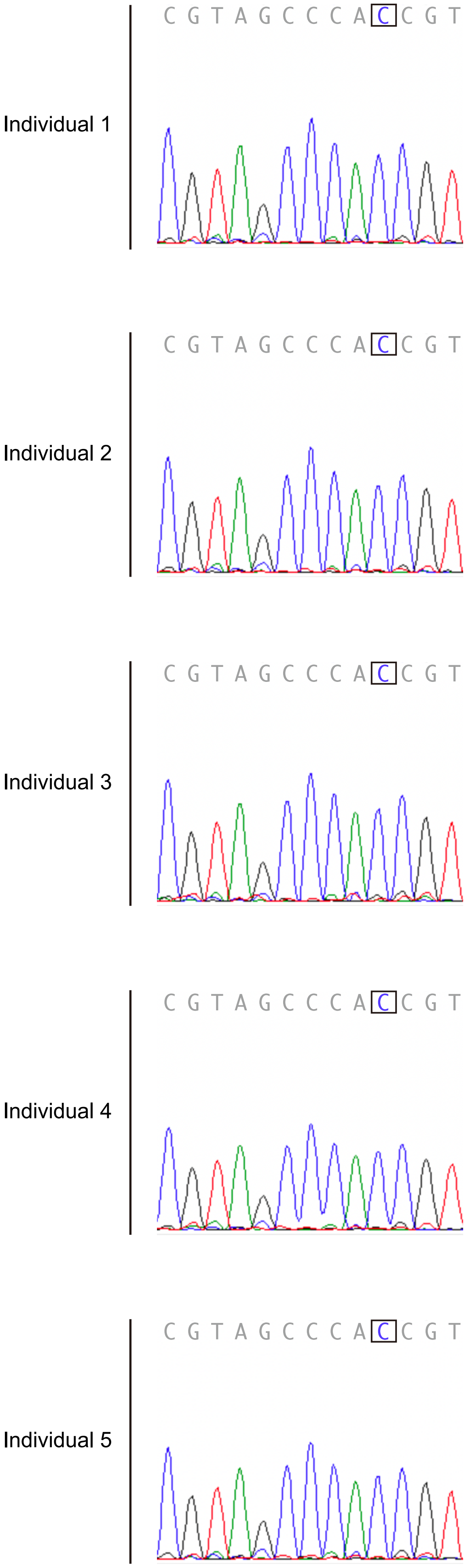

Fig. S6 Genotyping of SNP rs140124801 by the dideoxy sequencing. Representative electropherograms of five Individuals are shown: 1, C/C homozygote; 2, C/C homozygote; 3, C/C homozygote; 4, C/C homozygote;
