# Supplementary material for: Rapid detection of single nucleotide polymorphisms using the MinION nanopore sequencer: a feasibility study for perioperative precision medicine
Source: JA Clin Rep. 2022 Mar 4;8:17. doi: 10.1186/s40981-022-00506-7 (PMC8897523; doi:10.1186/s40981-022-00506-7)
Supplement: Supplementary file 4 — Additional file 4: Table S2. Statistics of the nanopore sequencing data. [file 40981_2022_506_MOESM4_ESM.pdf]

Table S2 Statistics of the nanopore sequencing data

SNP		Avg (bp)	Q score
rs1045642	Individual 1	414.5	12.5
	Individual 2	428.1	12.5
	Individual 3	416.4	12.7
	Individual 4	418.4	11.5
	Individual 5	429.0	9.4
rs1799971	Individual 1	714.2	11.8
	Individual 2	733.7	12.2
	Individual 3	723.3	12.0
	Individual 4	726.0	11.1
	Individual 5	723.9	9.2
rs2165870	Individual 1	921.0	9.5
	Individual 2	929.3	13.2
	Individual 3	891.6	13.0
	Individual 4	906.0	12.0
	Individual 5	929.1	9.5
rs4369876	Individual 1	432.2	14.8
	Individual 2	444.8	13.5
	Individual 3	434.6	13.3
	Individual 4	436.7	12.1
	Individual 5	440.0	11.7
rs33985936	Individual 1	463.7	13.8
	Individual 2	471.9	12.4
	Individual 3	464.3	13.9
	Individual 4	460.4	13.8
	Individual 5	467.5	10.9
rs140124801	Individual 1	482.4	13.3
	Individual 2	480.3	13.3
	Individual 3	477.0	13.3
	Individual 4	481.8	13.4
	Individual 5	474.0	12.0

Statistics of 1000 nanopore sequencing reads used for SNP genotyping.

Avg: average read length, Q score: average Phred quality score.
