# Supplementary material for: Rapid detection of single nucleotide polymorphisms using the MinION nanopore sequencer: a feasibility study for perioperative precision medicine
Source: JA Clin Rep. 2022 Mar 4;8:17. doi: 10.1186/s40981-022-00506-7 (PMC8897523; doi:10.1186/s40981-022-00506-7)
Supplement: Supplementary file 5 — Additional file 5: Tables S3-S8. Allele frequencies for each SNP as determined by nanopore sequencing. [file 40981_2022_506_MOESM5_ESM.pdf]

Table S3 Allele frequencies for rs1045642 determined by nanopore sequencing

	Read count				Allele frequency (%)		
Genotype	A	G	C/T	Total	A	G	C/T
Individual 1	994	5	1	1000	99.4	0.5	0.1
Individual 2	998	1	1	1000	99.8	0.1	0.1
Individual 3	1	998	1	1000	0.1	99.8	0.1
Individual 4	464	530	6	1000	46.4	53.0	0.6
Individual 5	463	537	0	1000	46.3	53.7	0

Table S4 Allele frequencies for rs1799971 determined by nanopore sequencing

	Read count				Allele frequency (%)		
Genotype	A	G	C/T	Total	A	G	C/T
Individual 1	446	554	0	1000	44.6	55.4	0
Individual 2	993	7	0	1000	99.3	0.7	0
Individual 3	435	565	0	1000	43.5	56.5	0
Individual 4	989	8	3	1000	98.9	0.8	0.3
Individual 5	994	6	0	1000	99.4	0.6	0

Table S5 Allele frequencies for rs2165870 determined by nanopore sequencing

	Read count				Allele frequency (%)		
Genotype	A	G	C/T	Total	A	G	C/T
Individual 1	5	985	10	1000	0.5	98.5	1.0
Individual 2	5	990	5	1000	0.5	99.0	0.5
Individual 3	488	507	5	1000	48.8	50.7	0.5
Individual 4	498	498	4	1000	49.8	49.8	0.4
Individual 5	580	416	4	1000	58.0	41.6	0.4

Table S6 Allele frequencies for rs4369876 determined by nanopore sequencing

	Read count				Allele frequency (%)		
Genotype	C	A	G/T	Total	C	A	G/T
Individual 1	981	0	19	1000	98.1	0	1.9
Individual 2	461	530	9	1000	46.1	53.0	0.9
Individual 3	979	0	21	1000	97.9	0	2.1
Individual 4	971	0	29	1000	97.1	0	2.9
Individual 5	943	0	57	1000	94.3	0	5.7

Table S7 Allele frequencies for rs33985936 determined by nanopore sequencing

	Read count				Allele frequency (%)		
Genotype	C	T	A/G	Total	C	T	A/G
Individual 1	992	8	0	1000	99.2	0.8	0.0
Individual 2	990	10	0	1000	99.0	1	0.0
Individual 3	994	6	0	1000	99.4	0.6	0.0
Individual 4	992	8	0	1000	99.2	0.8	0.0
Individual 5	993	7	0	1000	99.3	0.7	0.0

Table S8 Allele frequencies for rs140124801 determined by nanopore sequencing

	Read count				Allele frequency (%)		
Genotype	C	T	A/G	Total	C	T	A/G
Individual 1	998	1	1	1000	99.8	0.1	0.1
Individual 2	1000	0	0	1000	100	0	0
Individual 3	996	4	0	1000	99.6	0.4	0
Individual 4	999	1	0	1000	99.9	0.1	0
Individual 5	998	1	1	1000	99.8	0.1	0.1
